# High-Performance Multi-Level Grayscale Conversion by Driving Waveform Optimization in Electrowetting Displays

**DOI:** 10.3390/mi15010137

**Published:** 2024-01-16

**Authors:** Wanzhen Xu, Zichuan Yi, Mouhua Jiang, Jiashuai Wang, Zhengxing Long, Liming Liu, Feng Chi, Li Wang, Qiming Wan

**Affiliations:** 1 School of Electronic Information, University of Electronic Science and Technology of China, Zhongshan Institute, Zhongshan 528402, China; 2021024102@m.scnu.edu.cn (W.X.); 2022024132@m.scnu.edu.cn (M.J.); 202221021120@std.uestc.edu.cn (J.W.); mikaellzx@163.com (Z.L.); liulmxps@126.com (L.L.); chifeng@semi.ac.cn (F.C.); 2 South China Academy of Advanced Optoelectronics, South China Normal University, Guangzhou 510006, China; 3 School of Information and Optoelectronic Science and Engineering, South China Normal University, Guangzhou 510006, China; 4 School of Information Engineering, Zhongshan Polytechnic, Zhongshan 528400, China; wangli@zspt.edu.cn (L.W.); wanqiming@zspt.edu.cn (Q.W.)

**Keywords:** electrowetting display, multi-level grayscale, grayscale conversion

## Abstract

As a new type of reflective display, electrowetting display (EWD) has excellent dynamic display performance, which is based on polymer coatings. However, there are still some issues which can limit its performance, such as oil backflow and the hysteresis effect which reduces the stability and response speed of EWDs. Therefore, an effective driving waveform was proposed to overcome these drawbacks, which consisted of grayscale conversions between low gray levels and high gray levels. In the driving waveform, to stabilize the EWD at any initial grayscale (low gray levels/high gray levels), an exponential function waveform and an AC signal were used. Then, the grayscale conversion was performed by using an AC signal with a switching voltage to quickly achieve the target grayscale. Finally, another AC signal was used to stabilize the EWD at the target grayscale. A set of driving waveforms in grayscale ranging across four levels was designed using this method. According to the experimental results, oil backflow and the hysteresis effect could be effectively attenuated by the proposed driving waveforms. During conversion, the response speed of EWDs was boosted by at least 9.37% compared to traditional driving waveforms.

## 1. Introduction

Electrowetting is a phenomenon that changes the wettability between two phases by applying a voltage between substrates, which could control the movement and shape of droplets. The conceptual roots of EW technology could be traced back to G. Beni in 1981 [1]. Compared to traditional electrophoretic displays, EWDs based on electrowetting technology had better performance, with the advantages of a fast response speed, wide viewing angle, and excellent readability in daylight [2,3,4,5,6]. However, some shortcomings, such as oil splitting [7,8,9], oil backflow [10,11], charge trapping [12,13,14], and the hysteresis effect [15,16], have a negative impact on EWD quality. The main reason for these multiple problems was the interaction between the structure of the EWD and its driving mechanism. Therefore, combining the EWD structure and driving mechanism to design the driving waveform is an effective way to improve the performance of EWDs [17,18,19,20,21,22,23].

In multi-level grayscale conversion processes, oil backflow and the hysteresis effect could be suppressed to improve the grayscale stability and response speed of EWDs. Oil backflow, caused by charge trapping which is a phenomenon of trapping charges in an insulating layer of EWD pixels, can directly reduce aperture ratio. In recent years, a proposal was made for a portable driving scheme capable of releasing capture charges through reverse polarity voltage, resulting in a successful realization of a 4-level grayscale dynamic video in an active matrix EWD [24]. Simultaneously, a driving waveform containing a reset signal was suggested for releasing captured charges [25], which could improve the aperture ratio. As for the hysteresis effect, some scholars have conducted research on this topic. It has been verified that the use of AC driving waveforms could reduce the hysteresis of the contact angle during a wetting process [26,27]. To resolve the hysteresis effect, an amplitude–frequency mixed modulation driving system was proposed, which could also improve the response speed of EWDs [28]. Furthermore, a multi-waveform adaptive driving scheme was proposed, which, compared with the square-wave driving waveform, could reduce the maximum hysteresis difference of the hysteresis curve and improve the stability of grayscales [29]. These exceptional driving waveforms offer valuable insights and inspiration for the design of driving waveforms.

In this paper, a combined driving waveform has been proposed to achieve high-performance multi-level grayscale conversions based on the analysis of the driving principle of EWDs. An exponential signal was applied to attenuate abrupt variations in the driving waveform, which could prevent oil splitting. An AC signal was employed to release trapped charges, leading to improvements in the stability of EWDs. In addition, an AC signal with a switching voltage was designed to increase the EWDs’ response speed.

## 2. Driving Mechanism of EWDs

**Figure 1:**
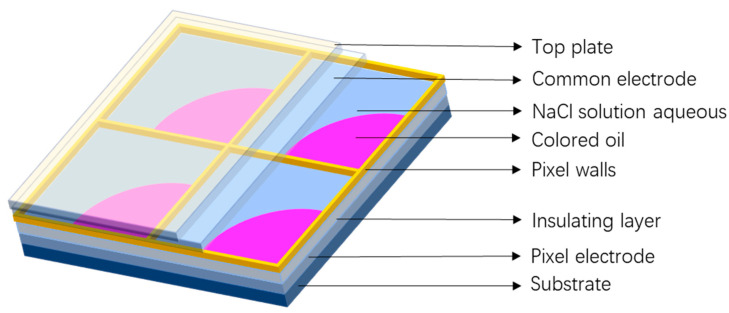
A three-dimensional schematic diagram of pixels in EWDs.

Each pixel of EWD is composed of a substrate, a pixel electrode, an insulating layer, a pixel wall, colored oil, NaCl solution, a common electrode, and a top plate [30,31,32,33,34,35,36,37,38]. Its structure is shown in Figure 1. The insulating layer is composed of a fluoropolymer Teflon AF 601S2 (DuPont, Wilmington, DE, USA). This material is highly soluble in fluorine-based solvents, which helps to prevent the electrolysis of liquids in EWDs. Additionally, to prevent oil from adhering to the pixel wall, it is coated with a hydrophilic and oleophilic fluoropolymer surface after plasma treatment. The upper and lower substrates are composed of ITO (indium tin oxide) glasses. The pixel contains oil made of an anthraquinone-type dye, an organic polymer with a high molar absorption coefficient and a wide spectral range. As for the EWD preparation, the process is as follows. Initially, a reticulated pixel wall is deposited on the panel by using photolithography, and then the panel is divided into micron-sized pixel grids, where the grid size is the size of an EWD’s pixel. After that, a non-polar colored oil is filled inside each pixel, and the color of oil matches the displayed color. Then, the electrolyte solution is filled as a second fluid and the common electrode. Finally, the substrate is placed and encapsulated by using a pressure-sensitive adhesive, thus completing the preparation of the EWD.

The driving process of EWDs is as follows: when the voltage applied between the two electrodes is below the threshold voltage, the insulating layer becomes hydrophobic, resulting in a flat spreading of colored oil in pixels. When the voltage between two electrodes is gradually increased, the interfacial tension presents changes, which further leads to a change in the contact angle, and the wettability of the insulating layer changes, resulting in an oleophobic property. In this way, the colored oil moves and deforms.

Equation (1) is the relationship between interfacial tension and contact angle. θ is the contact angle, $\gamma_{LG}$ is the interfacial tension between liquid and gas, $\gamma_{SG}$ is the interfacial tension between solid and gas, and $\gamma_{SL}$ is the interfacial tension between solid and liquid.

(1) $$\theta =arcos\frac{\gamma_{SG}-\gamma_{SL}}{\gamma_{LG}}$$

The Lippmann–Young equation can describe the changing process of the droplets’ contact angle on the solid interfacial with a driving voltage, as shown in Equation (2) [39].

(2) $$\cos\theta_{V}=\cos\theta_{0}+\frac{\varepsilon_{0}\varepsilon_{r}}{2d\gamma_{LG}}V^{2}$$

When the applied voltage is V, the contact angle becomes $\theta_{V}$. $\varepsilon_{0}$ is the vacuum dielectric constant, and $\varepsilon_{r}$ is the relative dielectric constant. d is the thickness of a hydrophobic insulation layer. From the above conclusion, it is evident that the application of voltage alters the contact angle of EWDs. Therefore, different effects can be achieved by varying different driving waveforms for driving EWDs.

## 3. Experimental Results and Discussion

### 3.1. Construction of the Experimental Platform

**Figure 2:**
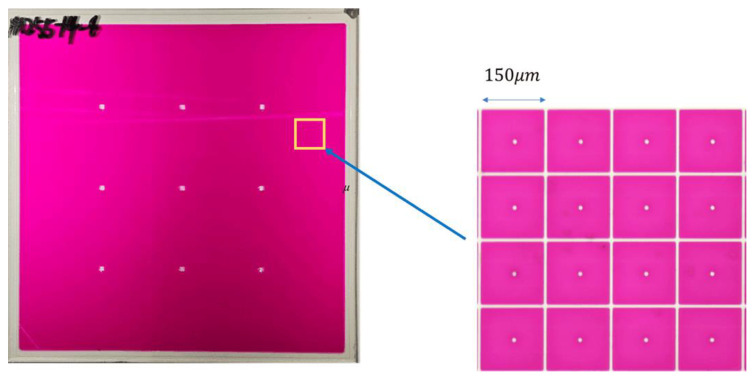
The EWD manufactured by us.

In this experiment, an integrated experimental platform was constructed. The platform consisted of a colorimeter, which could record the EWD’s luminance, and a voltage amplifier, which was used to amplify the driving voltage of driving waveforms [13]. In the experiment, the EWD used for testing was designed and manufactured by us, as shown in Figure 2. The oil color of the EWD was magenta. The panel size was 10 × 10 cm^2^ and the resolution was 320 × 240. The pixel size was 150 × 150 ${\mu m}^{2}$ The height of the pixel wall was 18 μm. The thickness of the insulating layer and the electrode plate was 1 nm and 2.5 μm, respectively.

### 3.2. Proposed Driving Waveforms

**Figure 3:**
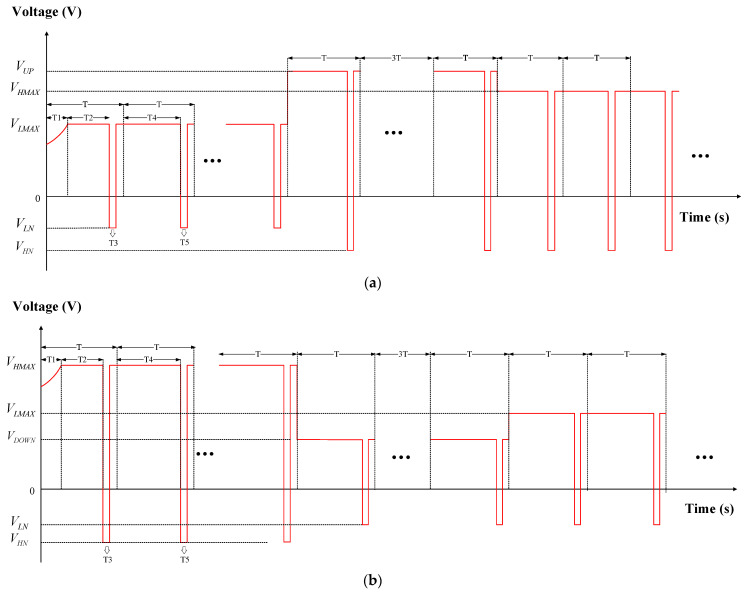
Diagrams of the proposed driving waveform. (**a**) The diagram of the conversion from low gray levels to high gray levels. (**b**) The diagram of the conversion from high gray levels to low gray levels.

To stably drive EWDs to achieve maximum luminance and a stable performance at a target grayscale, a driving waveform based on an exponential function signal and an AC signal was proposed in the initial driving stage. The exponential function signal could drive the EWDs swiftly. The AC signal was used to release tapped charges which could overcome oil backflow. At the same time, driving waveforms for grayscale conversions were also designed, as shown in Figure 3a,b.

As shown in Figure 3a, in the process of conversion from a low gray level to a high gray level, the driving waveform of the initial driving stage was also used to obtain an initial grayscale. In addition, a switching voltage was proposed to swiftly drive the EWDs from a low gray level to a high gray level. In this process, the switching voltage would be higher than the target voltage of the target grayscale, which could reduce the response time when the target grayscales were the same. In addition, the negative voltage could suppress oil backflow. The parameters of the driving waveform are explained in Table 1.

As shown in Figure 3b, in the process of conversion from a high gray level to a low gray level, the driving waveform of the initial driving stage was also used to obtain a high gray level. A switching voltage was proposed to solve the hysteresis effect and swiftly drive the EWDs from a high gray level to a low gray level. In this process, the switching voltage would be lower than the target voltage of the target grayscale, so as to reduce the response time when the target grayscales were the same. In addition, the negative voltage could suppress oil backflow. The parameters of the driving waveform are explained in Table 2.

### 3.3. Realization of Multi-Level Grayscales

**Figure 4:**
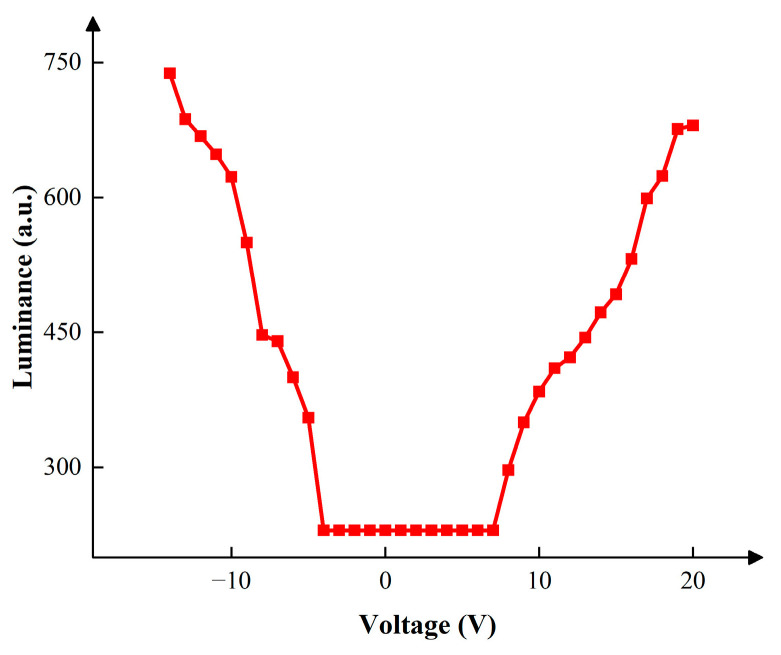
The luminance of the EWD when various DC driving voltages were applied. The luminance was 230 when the positive DC voltage was less than 8 V. The luminance was 230 when the negative DC voltage was less than 5 V. The maximum luminance was obtained when the DC voltage was 20 V.

**Figure 5:**
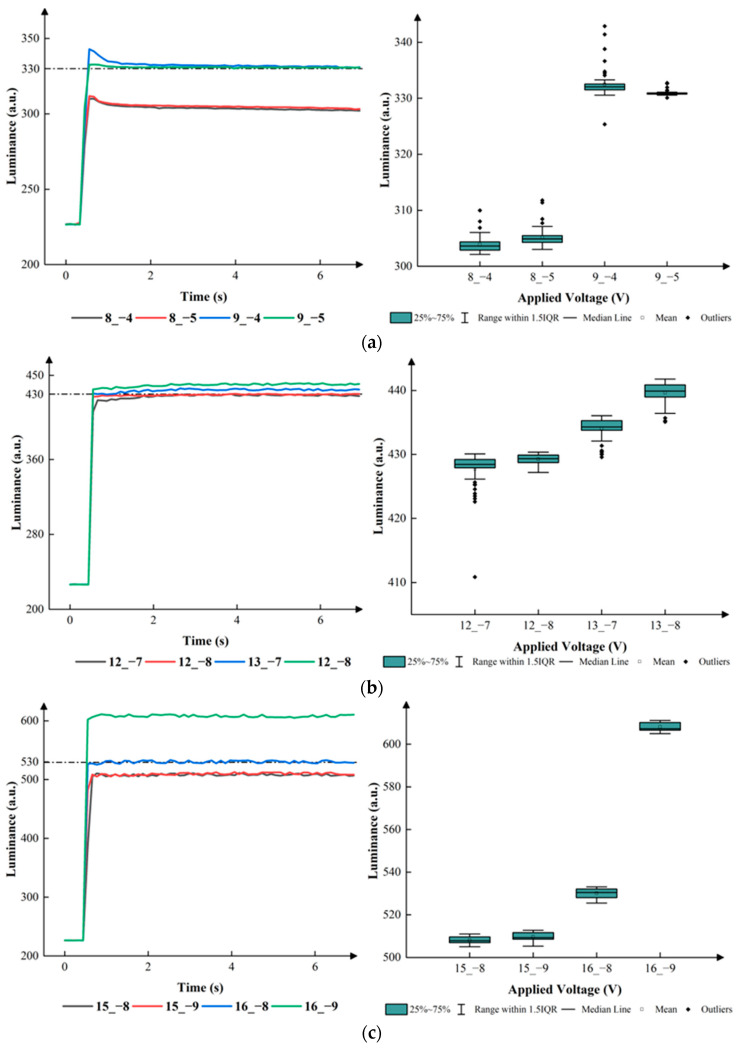
The EWD’s luminance when different voltages were applied. Assorted colors of curves represented different $V_{MAX}s$ and $V_{N}$s applied to EWDs. To take 8_−4 in (**a**) as an example, 8 is the value of $V_{MAX}$, and −4 is the value of $V_{N}$. (**a**) Luminance values and applied voltages in the first grayscale. (**b**) Luminance values and applied voltages in the second grayscale. (**c**) Luminance values and applied voltages in the third grayscale.

The corresponding relationship between the DC driving voltage and the luminance was measured as shown in Figure 4. The maximum luminance was 680 when 20 V positive DC voltage was applied. In this paper, the number of grayscales was set to four as an illustrative example, and the target luminance values of the four grayscales were set to 230, 330, 430, and 530. The target driving voltages were set to 0 V, 8–9 V, 12–13 V, and 15–16 V, respectively. Negative voltage $V_{N}$ was used to release trapped charges. The range of $V_{N}$ was set to 0 V, 4–5 V, 7–8 V and 8–9 V, respectively. The driving cycle T was set to 20 ms because 50 Hz is a frequency which could be perceived by human eyes. $T_{1}$ was set to 5 ms. $T_{2}$ was set to 10 ms to drive the EWD at the maximum luminance. $T_{3}$ was set to 0.5 ms to release trapped charges, which could prevent oil backflow caused by charge trapping. $T_{4}$ was set to 15 ms. $T_{5}$ was also set to 15 ms to release trapped charges. The relationship between EWDs’ luminance and $V_{MAX}$, $V_{N}$ in different grayscales is shown in Figure 5.

**Figure 6:**
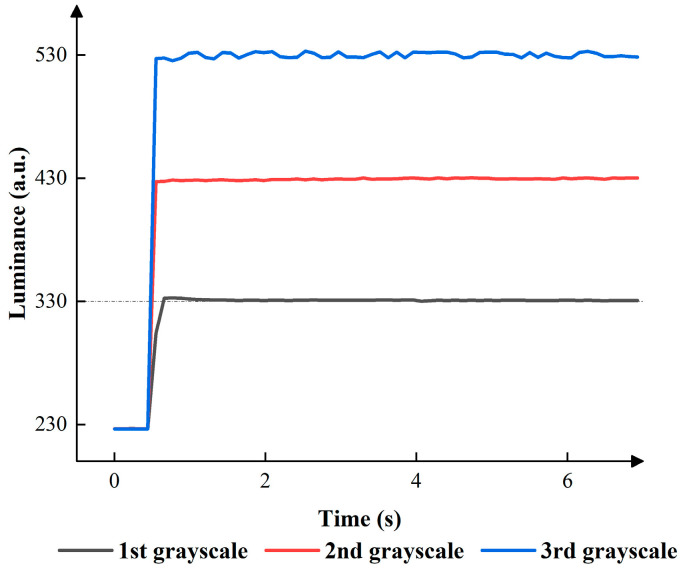
The luminance of the EWD when the best parameters were applied.

According to Figure 5, the applied positive and negative voltage could influence the luminance, and the best parameter of the initial driving stage could be obtained. In the first grayscale, the luminance remained between 304 and 305 when the applied positive voltage was 8 V. The luminance remained between 332 and 330 when the applied positive voltage was 9 V. The interquartile ranges (IQRs) were 4 and 1, when the applied negative voltage was −4 V and −5 V. In the second grayscale, the luminance remained between 428 and 430 when the applied positive voltage was 12 V. The luminance remained between 435 and 340 when the applied positive voltage was 13 V. The IQRs were 5 and 3, when the applied negative voltages were −7 V and −8 V. In the third grayscale, the luminance remained between 510 and 412 when the applied positive voltage was 15 V. The luminance remained between 530 and 610 when the applied positive voltage was 16 V. The IQR was 5 when the applied negative voltage was −8 V. Therefore, the best parameters could be obtained. $V_{MAX}$ was set to 9 V, 12 V, and 16 V in the first grayscale, the second grayscale, and the third grayscale, respectively. $V_{N}$ was set to −5 V, −8 V and −8 V in the first grayscale, the second grayscale, and the third grayscale, respectively. Figure 6 demonstrates the relationship between the driving time and the luminance of the EWD in the three grayscales.

### 3.4. Conversions from Low Gray Levels to High Gray Levels

**Figure 7:**
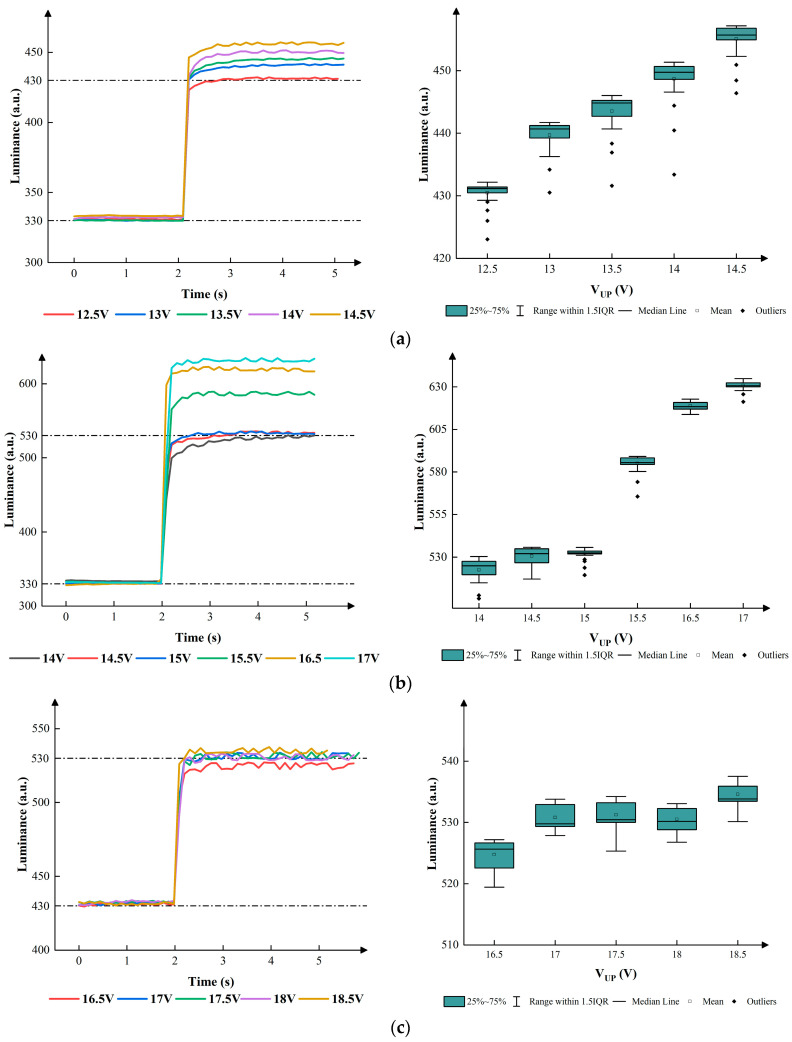
The luminance of EWDs when a low gray level converted to a gray level. (**a**) Conversion process from the first grayscale to the second grayscale when different $V_{UP}$s were applied. (**b**) Conversion process from the first grayscale to the third grayscale when different $V_{UP}$s were applied. (**c**) Conversion process from the second grayscale to the third grayscale when different $V_{UP}$s were applied.

Since no voltage needed to be applied to drive the 0-level grayscale, the conversion between the zeroth grayscale and the other grayscales was not considered in this section. In this section, the parameters shown in Figure 6 were used to achieve low gray levels and high gray levels. $V_{LMAX}$, $V_{HMAX}$, $V_{LN}$, and $V_{HN}$ were set to 9 V, 12 V, −5 V, and −8 V, respectively, when the first grayscale converted to the second grayscale. $V_{LMAX}$, $V_{HMAX}$, $V_{LN}$, and $V_{HN}$ were set to 9 V, 16 V, −5 V, and −8 V, respectively when the first grayscale converted to the third grayscale. The value of $V_{UP}$ was also evaluated to obtain the best performance. Figure 7 shows the relationship between the luminance and $V_{UP}$ in the different conversion processes within 5 s.

According to Figure 7, the change in luminance distribution was influenced by $V_{UP}$. In the process of conversion from the first grayscale to the second grayscale, the mean luminance values remained at 430, 440, 443, 448, and 457 when $V_{UP}$ was set to 12.5 V, 13 V, 13.5 V, 14 V, and 14.5 V, respectively. The IQRs were 3, 7, 7, 8, and 6. In the process of conversion from the first grayscale to the third grayscale, the mean luminance values remained at 620 and 627 when the $V_{UP}s$ were set to 16.5 V and 17 V. It is evident that the converted luminance was much higher than the target luminance, which led to an unstable luminance and had an adverse effect on the process of conversions. Therefore, the $V_{UP}s$ were set to 14 V, 14.5 V,15 V, and 15.5 V. The mean luminance values remained at 528, 530, 531, and 589 when the $V_{UP}s$ were set to 14 V, 14.5 V,15 V, and 15.5 V. The IQRs were 15, 18, 4, and 7. In the process of conversion from the second grayscale to the third grayscale, the mean luminance values remained at 525, 532, 533, 531, and 534 when $V_{UP}$ was set to 16.5 V, 17 V, 17.5 V, 18 V, and 18 V, respectively. The IQRs were 8, 6, 7, 5, and 4. Eventually, the best parameters could be obtained when the $V_{UP}$s were set to 12.5 V, 15 V, and 18 V in the three conversion processes.

### 3.5. Conversions from High Gray Levels to Low Gray Levels

**Figure 8:**
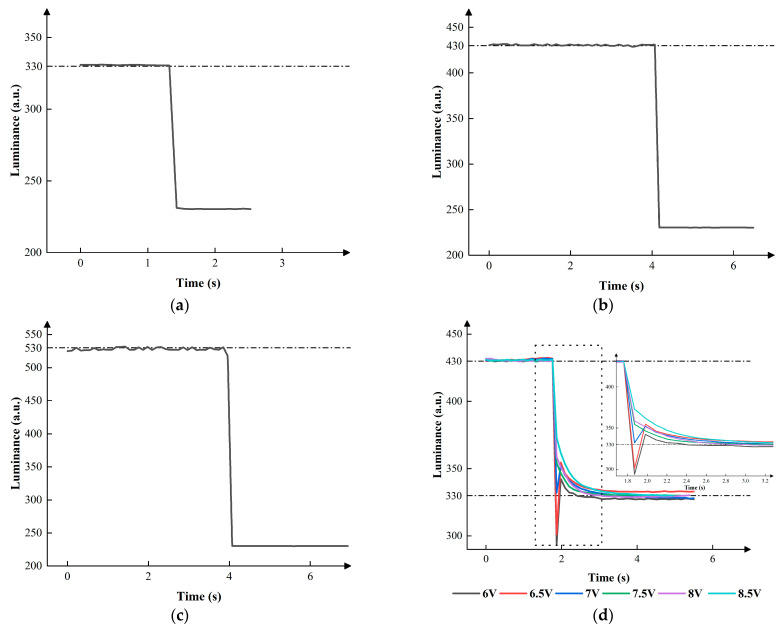

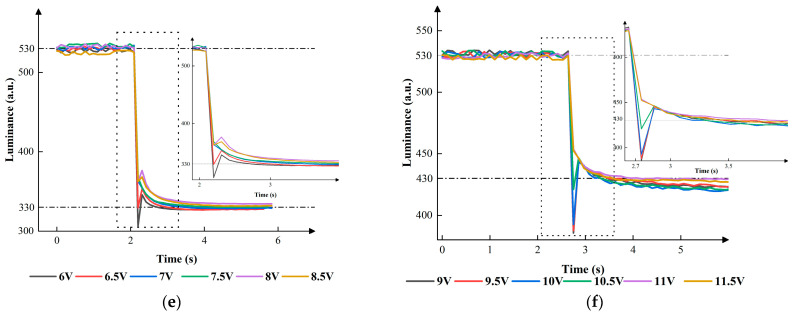
The luminance of EWDs when a low gray level converted to a high gray level. (**a**) Conversion process from the first grayscale to the zeroth grayscale. (**b**) Conversion process from the second grayscale to the zeroth grayscale. (**c**) Conversion process from the third grayscale to the zeroth grayscale. (**d**) Conversion process from the second grayscale to the first grayscale when different $V_{DOWN}$s were applied. (**e**) Conversion process from the third grayscale to the first grayscale when different $V_{DOWN}$s were applied. (**f**) Conversion process from the third grayscale to the second grayscale when different $V_{DOWN}$s were applied.

During the conversion from high gray levels to the zeroth grayscale, there was no need to design a conversion driving waveform, because no voltage was applied in this process. As for other conversion processes, the driving waveforms and parameters were tested. $V_{LMAX}$, $V_{HMAX}$, $V_{LN}$, and $V_{HN}$ were set to 9 V, 12 V, −5 V, and −8 V, respectively, when the second grayscale converted to the first grayscale. $V_{LMAX}$, $V_{HMAX}$, $V_{LN}$, and $V_{HN}$ were set to 9 V, 12 V, −5 V, and −8 V, respectively, when the third grayscale converted to the first grayscale. $V_{LMAX}$, $V_{HMAX}$, $V_{LN}$, and $V_{HN}$ were set to 9 V, 16 V, −5 V, and −8 V, respectively, when the third grayscale converted to the second grayscale. The value of $V_{DOWN}$ was also evaluated to obtain the best performance. Figure 8 shows the relationship between the luminance and $V_{DOWN}$ in the different conversion processes.

According to Figure 8, $V_{DOWN}$ could affect the performance of EWDs. In the processes of conversion from other grayscales to the zeroth grayscale, the luminance nearly descended to 230. In the process of conversion from the second grayscale to the first grayscale, the luminance first swiftly dropped from 430 to less than 300, then rose to between 330 and 350, and finally stabilized at 330 when $V_{DOWN}$ was set to 6 V or 6.5 V. The luminance first swiftly dropped from 430 to about 335, then rose to 350, and finally stabilized at 330 when $V_{DOWN}$ was set to 7 V. The luminance first swiftly dropped from 430, then dropped slowly, and finally stabilized at 330 when $V_{DOWN}$ was set to 7.5 V, 8 V, or 8.5 V. In the process of conversion from the third grayscale to the first grayscale, the luminance first swiftly dropped from 530 to less than 330, then rose to 345, and finally stabilized at 330 when $V_{DOWN}$ was set to 6 V. The luminance first swiftly dropped from 530 to about 330, then rose to 353, and finally stabilized at 330 when $V_{DOWN}$ was set to 6.5 V. The luminance first swiftly dropped from 530, then slowly dropped, and finally stabilized at 330 when $V_{DOWN}$ was set to 7 V, 7.5 V, or 8 V. The luminance first swiftly dropped from 530, then slowly increased a little, and finally stabilized at 330 when $V_{DOWN}$ was set to 8 V or 8.5 V. In the process of conversion from the third grayscale to the second grayscale, the luminance first swiftly dropped from 530 to less than 400, then rose to about 440, and finally slowly stabilized when $V_{DOWN}$ was set to 9 V, 9.5 V, 10 V, or 10.5 V. The luminance first swiftly dropped from 530 to about 330, then slowly dropped, and finally stabilized at 330 when $V_{DOWN}$ was set to 7 V. In summary, switching voltage could reduce the hysteresis effect, resulting in a faster grayscale conversion. Eventually, the best parameters could be obtained when $V_{DOWN}$s were set to 7.5 V, 7.5 V, and 11 V in the three processes of conversion.

### 3.6. Performance of the Proposed Driving Waveform

**Figure 9:**
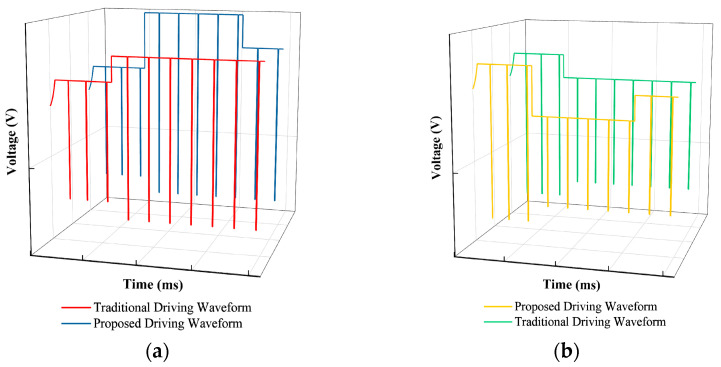
The proposed driving waveform and the traditional driving waveform in a grayscale conversion process. (**a**) Driving waveforms from a low gray level to a high gray level. (**b**) Driving waveforms from a high gray level to a low gray level.

Traditional driving waveforms were used to contrast the performance of the proposed driving waveform. Compared to the proposed driving waveform, the traditional driving waveform did not use $V_{UP}$ and $V_{DOWN}$ in the process of conversion. The luminance curves of the traditional driving waveform and the proposed driving waveform are shown in Figure 9.

**Figure 10:**
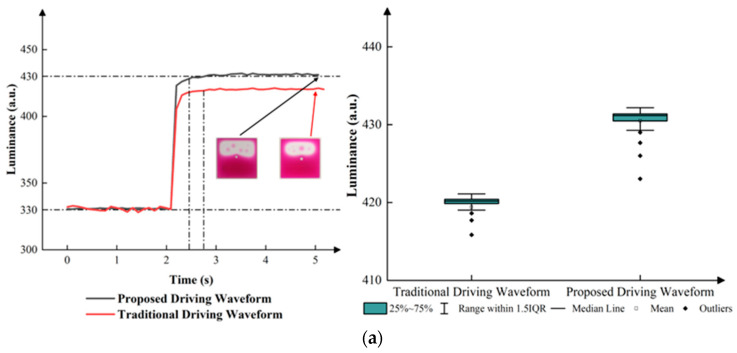

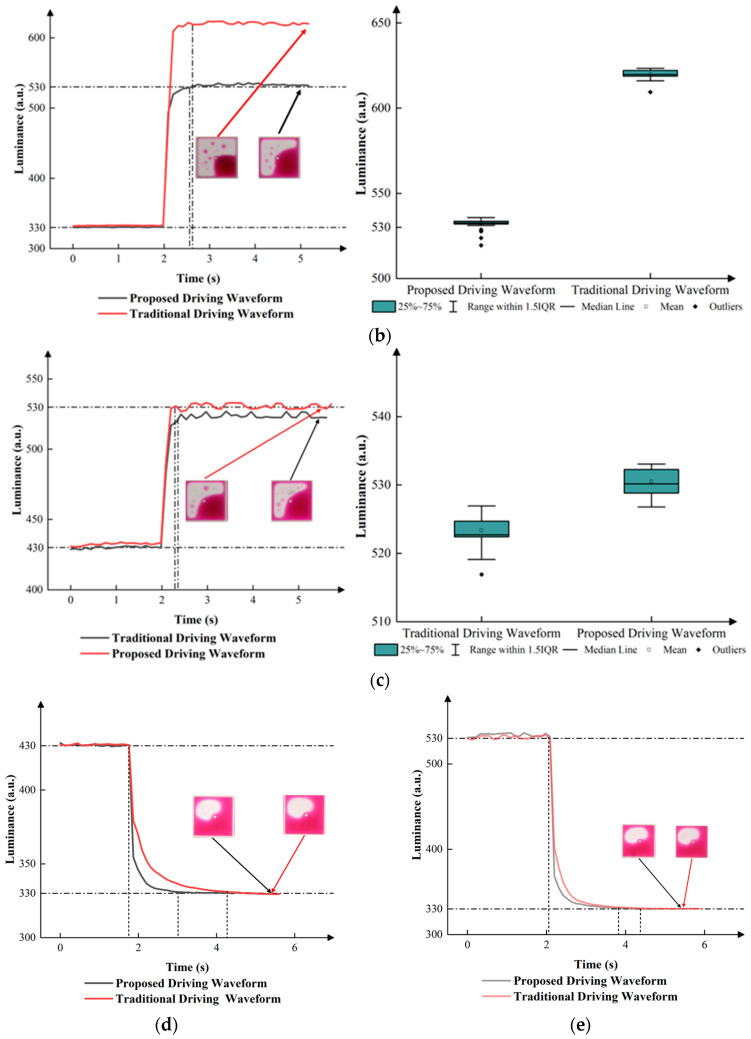

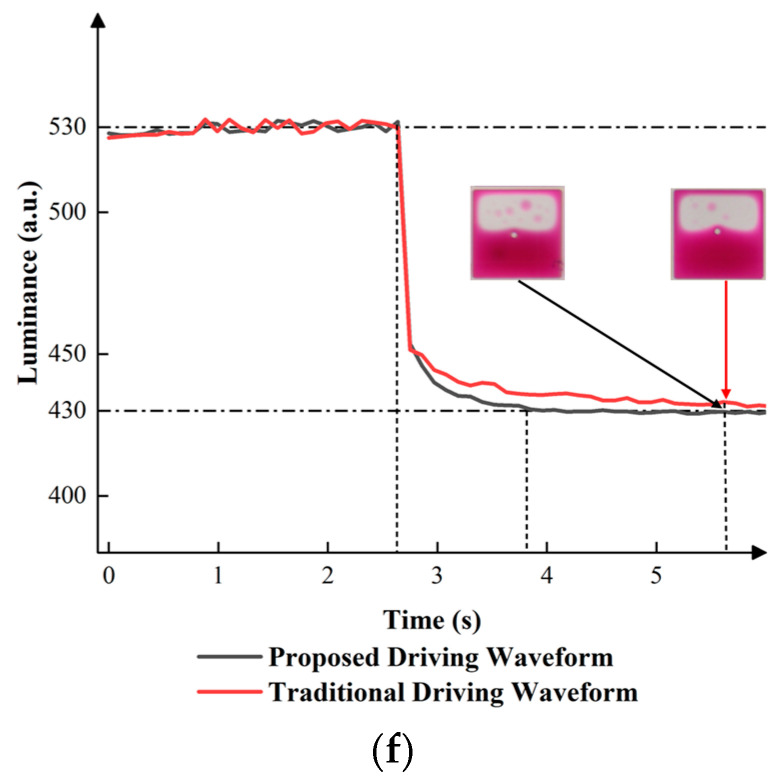
Luminance curves of the proposed driving waveform and the traditional driving waveform in different processes of conversion. (**a**) Luminance curves from the first grayscale to the second grayscale. (**b**) Luminance curves from the first grayscale to the third grayscale. (**c**) Luminance curves from the second grayscale to the third grayscale (**d**) Luminance curves from the second grayscale to the first grayscale. (**e**) Luminance curves from the third grayscale to the first grayscale. (**f**) Luminance curves from the third grayscale to the second grayscale.

According to Figure 10, compared to the traditional driving waveform, the proposed driving waveform had a better performance. In the process of conversion from the first grayscale to the second grayscale, the luminance of the proposed driving waveform increased from 330 to 430 and remained at 430, while the luminance of the traditional driving waveform always remained at 420. In addition, the proposed driving waveform had a shorter response time. In the process of conversion from the first grayscale to the third grayscale, the luminance of the proposed driving waveform increased from 330 to 530 and remained at 530, while the luminance of the traditional driving waveform could remain at over 600. In addition, the proposed driving waveform had a shorter response time. In the process of conversion from the second grayscale to the third grayscale, the luminance of the proposed driving waveform increased from 430 to 530 and remained at 530, while the luminance of the traditional driving waveform always remained at about 525. Apart from this, the proposed driving waveform had a shorter response time. In the process of conversion from the second grayscale to the first grayscale, the luminance decreased from 430 to 330 and remained at 330, while the proposed driving waveform had a shorter response time. In the process of conversion from the third grayscale to the first grayscale, the luminance decreased from 530 to 330 and remained at 330, while the proposed driving waveform had a shorter response time. In the process of conversion from the third grayscale to the second grayscale, although the luminance decreased from 530 to 430 and remained at 430, the proposed driving waveform had a shorter response time. Overall, the proposed driving waveform decreased the response time of EWDs, resulting in an improved performance, as shown in Table 3.

## 4. Conclusions

In order to improve the response speed and stability in the conversion process of multi-level grayscales in EWDs, a new combined driving waveform was proposed in this paper, which was based on an exponential function and AC signals. Compared to the traditional driving waveform, the application of the proposed driving waveform could be a helpful solution to suppress the oil backflow and hysteresis effects for EWDs during the conversion of the four-level grayscale, which could provide a certain research direction for the design of driving waveforms and promote the progress of paper-like display technology.

## Figures and Tables

**Table 1:** Parameters of the driving waveform.

Parameter	Description
$V_{LMAX}$	The target driving voltage which could drive the pixel to a low gray level
$V_{LN}$	A negative voltage which could release trapped charges
$V_{HMAX}$	The target driving voltage which could drive the pixel to a high gray level
$V_{HN}$	A negative voltage which could release trapped charges
$V_{UP}$	A switching voltage which could drive the pixel swiftly from a low gray level to a high gray level
T	The duration of a driving cycle
$T_{2}$, $T_{4}$	The duration of the DC driving process
$T_{3}$, $T_{5}$	The duration of the negative voltage

**Table 2:** Parameters of the driving waveform.

Parameter	Description
$V_{LMAX}$	The target driving voltage which could drive the pixel to a low gray level
$V_{HMAX}$	The target driving voltage which could drive the pixel to a high gray level
$V_{HN}$	A negative voltage which could release trapped charges
$V_{LN}$	A negative voltage which could release trapped charges
$V_{DOWN}$	A switching voltage which could drive the pixel swiftly from a high gray level to a low gray level
T	The duration of a driving cycle

**Table 3:** Performance of the proposed driving waveform.

Initial Gray Level	Target Gray Level	Response Speed Improvement Ratio
1	2	46.28%
1	3	9.37%
2	3	17.29%
2	1	49.6%
3	1	24.14%
3	2	60.53%

## Data Availability

Data are contained within the article.
